# Nutritional Interventions and Acupuncture‐Based Strategies for Exercise‐Induced Fatigue and Recovery: Mechanisms and Integrative Perspectives

**DOI:** 10.1002/fsn3.71871

**Published:** 2026-05-15

**Authors:** Yaqin Yang, Yao Yao, Weiyu Lu, Jiazhou Li, Lijuan Zou, Huashan Pan

**Affiliations:** ^1^ Institute of Chinese Medicine Health Care Guangdong Food and Drug Vocational College Guangzhou Guangdong China; ^2^ Clinical Medical College of Acupuncture Moxibustion and Rehabilitation Guangzhou University of Chinese Medicine Guangzhou Guangdong China; ^3^ The Eighth Affiliated Hospital Sun Yat‐Sen University Shenzhen Guangdong China; ^4^ Guangdong Chaozhou Health Vocational College Chaozhou Guangdong China; ^5^ School of Nursing Guangdong Food and Drug Vocational College Guangzhou Guangdong China; ^6^ Dongguan Institute of Guangzhou University of Chinese Medicine Dongguan Guangdong China

**Keywords:** acupuncture, electroacupuncture, exercise‐induced fatigue, mitochondrial function, nutritional interventions, oxidative stress

## Abstract

Exercise‐induced fatigue (EIF) refers to a reduced capacity to sustain a specified level or intensity of physical activity and is generally regarded as a physiological warning signal following excessive exercise rather than a pathological state. It involves peripheral disturbances related to energy depletion, metabolite accumulation, oxidative stress, inflammation, and mitochondrial dysfunction, together with central changes involving neuroendocrine networks and the gut–brain axis. In this review, we synthesize evidence from nutritional interventions and acupuncture‐based strategies and outline a conceptual mechanistic framework relevant to EIF management. Nutritional approaches include macronutrient manipulation, vitamins, plant‐derived polyphenols and polysaccharides, traditional herbal extracts, probiotics, and fermented products. Acupuncture, electroacupuncture (EA), and transcutaneous electrical acupoint stimulation (TEAS) have been associated with regulation of autonomic function and hypothalamic–pituitary–adrenal axis activity, modulation of pain and affective circuits, and changes in skeletal muscle perfusion, mitochondrial function, and antioxidant and anti‐inflammatory signaling. We highlight partially overlapping biological targets, including the AMPK/PGC‐1α axis, Nrf2 and NF‐κB signaling pathways, and cytokine networks, and propose a conceptual model in which nutritional interventions are primarily linked to substrate availability and redox support, whereas acupuncture‐based strategies are primarily linked to neuroendocrine and inflammatory regulation, with both being associated with mitochondrial function and recovery‐related processes. Current evidence is limited by protocol heterogeneity, small sample sizes, sparse mechanistic biomarker profiling, and a predominance of animal studies. We therefore outline priorities for multi‐arm randomized controlled trials, standardization of EA/TEAS parameters, multi‐omics integration, and the development of individualized prescription models. Shared mechanistic features between nutritional and acupuncture‐based interventions may inform future hypothesis‐driven studies of EIF; however, whether combined use provides additive or synergistic clinical benefit remains unproven and requires rigorous validation.

## Graphical abstract

Excessive exercise induces exercise‐induced fatigue through multiple interconnected mechanisms, including energy shortage and metabolite accumulation, oxidative stress and inflammation, mitochondrial dysfunction, and neuroendocrine strain. Integrative management strategies include nutrition‐based approaches and acupuncture‐based interventions. Nutrition‐based strategies, such as carbohydrate/protein timing, polyphenols and herbal extracts, and probiotics or fermented foods, mainly support fuel availability, buffer oxidative stress, and modulate gut‐related signaling. Acupuncture‐based strategies, including acupuncture, electroacupuncture, and transcutaneous electrical acupoint stimulation, primarily regulate autonomic and stress responses, influence pain and tissue perfusion, and dampen inflammatory signaling. These two approaches converge on shared pathways involving energy sensing, antioxidant defense, inflammatory control, and mitochondrial efficiency, and may help reduce fatigue burden, accelerate recovery after heavy exercise, and regulate both central and peripheral fatigue.

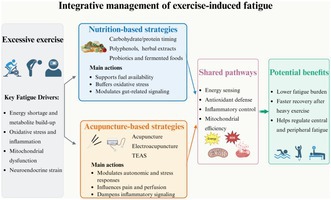

## 1 Introduction

Exercise‐induced fatigue (EIF) is a reversible decline in the ability to sustain a given exercise intensity or power output under a defined workload. Rather than a single biochemical event, EIF reflects the breakdown of multi‐organ and multi‐system homeostasis, integrating peripheral and central processes, as emphasized by recent narrative and mechanistic works (Yu et al. 2025; Zhao et al. 2026; Zhou et al. 2023). It is important to emphasize that EIF may serve as a self‐regulatory physiological signal under certain conditions; elucidating its underlying mechanisms will help us to better control and harness EIF, particularly in athletes (Cordeiro et al. 2017). Central fatigue originates in the central nervous system and is defined as a failure of the central nervous system to adequately drive the musculature; it may be related to depletion or accumulation of neurotransmitters such as serotonin (5‐hydroxytryptamine) and dopamine. Comprehensive descriptions of the mechanisms underlying central fatigue can be found in recent reviews (Zhou et al. 2023).

With respect to peripheral fatigue, EIF mechanisms can be grouped into interconnected domains, including energy depletion and accumulation of metabolites, oxidative stress and inflammation, and mitochondrial dysfunction and impaired quality control (Yu et al. 2025). Briefly, during sustained or high‐intensity exercise, muscle and liver glycogen stores become depleted and ATP resynthesis cannot match ATP hydrolysis. In parallel, lactate, inorganic phosphate, and H^+^ accumulate, impairing excitation–contraction coupling and cross‐bridge cycling (Davis 1995; Yu et al. 2025). Reactive oxygen species (ROS) accumulation during exercise triggers lipid peroxidation, protein and DNA damage, can disrupt the tricarboxylic acid cycle and even initiate apoptosis. ROS‐dependent activation of NF‐κB and related inflammatory pathways promotes systemic inflammation and aggravates muscle dysfunction (Chen et al. 2021; Kim et al. 2022; Liu et al. 2018). In addition, reduced oxidative phosphorylation capacity, loss of mitochondrial membrane potential, and accumulation of damaged mitochondria compromise ATP supply and exacerbate ROS generation (Powers et al. 2020; Yu et al. 2025).

Non‐steroidal anti‐inflammatory drugs (NSAIDs), such as ibuprofen and paracetamol, are widely used to increase pain tolerance and reduce inflammation from injuries (Lundberg and Howatson 2018). However, the effects of NSAIDs in alleviating EIF are inconsistent (Lima et al. 2016; Roberts et al. 2024). Recent studies have demonstrated that analgesic drugs can attenuate long‐term gains in muscle mass and strength in young healthy individuals (Lundberg and Howatson 2018). In addition, NSAIDs are associated with an increased risk of adverse gastrointestinal, renal, and cardiovascular effects, and with interference with adaptive training responses (Bacchi et al. 2012). Thus, pharmacological control of EIF appears suboptimal at present. Against this mechanistic and therapeutic background, non‐pharmacological strategies are attractive, especially nutritional interventions and acupuncture‐based approaches. For nutritional interventions, beyond classical explanations (substrate depletion, metabolite accumulation, oxidative stress), an increasing body of evidence indicates that the gut microbiota is a key regulator linking energy metabolism, immune–inflammatory responses, and EIF (Dmytriv et al. 2024; Zhao et al. 2026). Through the “gut–muscle” and “gut–brain” axes, microbial communities and their metabolites influence exercise performance, recovery, and fatigue perception (Dohnalová et al. 2022; Zhou et al. 2023). At the same time, acupuncture‐based strategies rooted in traditional Chinese medicine (TCM) have gained increasing attention in sports medicine and fatigue‐related conditions as safe, drug‐free interventions, capable of rapid neuroendocrine and inflammatory modulation (Kang et al. 2021; Liu et al. 2022; Wang et al. 2017).

Despite increasing interest in non‐pharmacological strategies for EIF, current research remains largely fragmented, with nutritional and acupuncture‐based interventions typically investigated in isolation. Moreover, although both approaches have been shown to modulate key processes such as energy metabolism, oxidative stress, inflammation, and neuroendocrine regulation, a systematic integration of their shared and potentially complementary mechanisms is lacking. This gap limits our ability to develop coordinated, mechanism‐based intervention strategies for EIF. Therefore, the objectives of this review are threefold: (1) to synthesize current evidence on nutritional and acupuncture‐based interventions in EIF; (2) to identify potential convergent biological targets and shared signaling pathways; and (3) to propose an integrative mechanistic framework that conceptualizes their possible complementarity in regulating mitochondrial function, redox balance, and neuroendocrine–immune interactions. Throughout, we distinguish direct EIF evidence from indirect or extrapolative evidence derived from related fatigue conditions or non‐EIF disease models. Importantly, we critically evaluate the current level of evidence and highlight the absence of direct clinical data supporting synergistic effects, thereby outlining priorities for future translational research.

## 2 Nutritional Interventions in EIF

### 2.1 Nutritional Strategies Targeting Energy Depletion

Carbohydrates (CHO) are important substrates for contracting muscle during prolonged exercise; fatigue is often associated with muscle glycogen depletion and/or hypoglycemia (Costill and Hargreaves 1992). CHO and fluid intake before, during, and after exercise remain foundational for managing EIF. Observational and interventional studies show that inadequate hydration and insufficient CHO availability are associated with higher ratings of perceived exertion and premature fatigue during training and competition (Cesanelli et al. 2021; Costill and Hargreaves 1992). By sharply reducing CHO intake and increasing fat, ketogenic diets (KD) promote hepatic ketogenesis and markedly increase fat oxidation during exercise (Ma and Suzuki 2019). Narrative synthesis suggests that in some ultra‐endurance contexts, KD can reduce reliance on glycogen and delay “glycogen‐dependent” peripheral fatigue. However, evidence is heterogeneous, and several studies indicate impaired high‐intensity performance and peak power under KD, presumably due to constrained glycolytic flux and limited rapid ATP resynthesis (Ma and Suzuki 2019). Amino acid‐based strategies can modulate both energy provision and fatigue‐related signaling. In a double‐blind, randomized crossover trial, a cysteine/glutamine mixture increased fat oxidation and reduced subjective fatigue during submaximal endurance exercise in humans, consistent with a shift from CHO‐dominated metabolism toward more efficient fat utilization (Ma, Ono, et al. 2022). Studies in rodents and humans indicate that oat protein, β‐glucan‐rich polysaccharides, and composite formulas can increase muscle mass or strength, improve endurance performance, and mitigate fatigue‐related biochemical changes (Kim et al. 2023; Xu et al. 2013). In patients with chronic obstructive pulmonary disease‐associated cachexia, targeted medical nutrition high in energy, protein, and anti‐inflammatory lipids improved body weight and muscle function; however, these findings should be regarded as indirect evidence relevant to fatigue‐related nutritional support rather than direct EIF evidence (Calder et al. 2018). Besides, Ma et al. (2021) systemically reviewed other nutrient supplements targeting energy insufficiency‐mediated fatigue, including dietary ATP, phosphocreatine, glucose, piperine, and carnitine.

Nutrient timing is a popular nutritional strategy that involves the consumption of nutrients in and around an exercise session. The post‐exercise period is widely considered the most critical, because it is considered to maximize exercise‐induced muscular adaptations and facilitate repair of damaged tissue (Kerksick et al. 2017). Researchers found that co‐ingestion of protein and amino acid stimulates muscle protein synthesis and optimizes whole body protein balance compared with the intake of carbohydrate only (Koopman et al. 2005). The concept of a narrow “post‐exercise anabolic window” is debated, but nutrient timing clearly influences EIF. Evidence supports CHO and amino acid ingestion 2–3 h before exercise, appropriate CHO–electrolyte intake during prolonged sessions, and timely post‐exercise recovery nutrition to facilitate glycogen resynthesis and reduce fatigue (Aragon and Schoenfeld 2013; Cesanelli et al. 2021; Ma, Ono, et al. 2022). However, increased complexity in dosing, interactions, and inter‐individual variability should be considered. High‐quality trials comparing timing and combination regimens specifically with EIF endpoints are still limited and should be prioritized.

### 2.2 Oxidative Stress and Inflammation: Antioxidants and Bioactive Components

Classical antioxidants such as vitamins C and E have long been used by athletes to prevent exercise‐induced muscle damage. A recent meta‐analysis reported that low‐dose vitamin E supplementation (< 500 IU/day) significantly reduced post‐exercise creatine kinase and markers of oxidative stress, particularly in trained individuals (Kim et al. 2022). However, whether acute or chronic supplementation with vitamins C and E could produce a beneficial effect on physical performance is still inconclusive. And accumulating evidence suggests that high doses or chronic administration of antioxidant vitamins can blunt training‐induced mitochondrial and metabolic adaptations, likely by excessively suppressing ROS‐dependent signaling (Kim et al. 2022; Ma et al. 2021). Similarly, oral glutathione is bioavailable and increases systemic antioxidant capacity, some studies demonstrated it can reduce exercise‐induced muscular injury and improve physical performance in athletes, but direct evidence of efficacy against EIF remains limited (Lee et al. 2023). Coenzyme Q10, a key electron carrier in the mitochondrial respiratory chain, has been reported to lower exercise‐induced oxidative and to increase time to fatigue in some trials, although findings are heterogeneous (Ma et al. 2021). Overall, antioxidant supplementation should be individualized and carefully dosed to balance protection from excessive oxidative injury against preservation of beneficial redox signaling.

Recently, attention has been shifted to the effects of nutraceutical bioactive compounds, such as polyphenols, polysaccharides, and glycosides. Studies have demonstrated that these bioactive compounds both exert a significant effect in exercise‐induced muscle damage and play a biological/physiological role in alleviating EIF and improving physical performance (Malaguti et al. 2013; Xie et al. 2026; Zhou and Jiang 2019). In addition, polyphenols from processed fresh‐cut pitaya (treated with ozone) showed better anti‐fatigue efficacy, evidenced by higher liver glycogen content, greater antioxidant capacity, and longer exhaustion time; highlighting food processing could influence bioactive profiles and biological effects (Li et al. 2025). *Lepidium meyenii* (Maca) and its characteristic macamides improved endurance capacity, limited mitochondrial‐mediated skeletal muscle damage, and modulated hormonal and neurochemical pathways, linking metabolic and neuroendocrine aspects of fatigue (Orhan et al. 2022; Zhu et al. 2021). Grape seed proanthocyanidins possessed the beneficial properties of anti‐inflammatory, antioxidant, and mitochondrial protection to improve endurance performance in mice (Liu et al. 2018). Gastrodin administration in a mouse forced swimming model reduced fatigue by preserving muscle and liver glycogen and increasing superoxide dismutase activity. These results revealed that gastrodin activated AMPK and Nrf2/HO‐1 pathways to improve performance in exhaustive exercise (Zhang et al. 2023). The trilobatin alleviated exhaustive EIF in mice by activating Nrf2/ARE signaling and inhibiting ferroptosis, leading to improved mitochondrial structure and reduced oxidative damage (Xiao et al. 2022). Similarly, the phlorizin protected against exercise‐induced oxidative stress through Nrf2/ARE activation and preservation of membrane ATPase activities, thereby exerting an anti‐fatigue effect (Ma, Deng, et al. 2022). These results collectively highlight an effective strategy for mitigating EIF: plant‐derived bioactive compounds attenuate apoptosis by modulating the Nrf2/ARE–ferroptosis axis, thereby alleviating EIF.

Natural oils and fermented products also show promise in anti‐fatigue properties. Camellia oil modulates antioxidant capacity, muscle fiber composition, and gut microbiota, thereby enhancing endurance in mice (Huang et al. 2024). Similar results have also been reported for citrus essential oils, which mitigated EIF in rats by promoting glucose‐dependent energy supply and inhibiting oxidative stress (Tian et al. 2022). Fermented deer blood and propolis ethanol extract have also been reported to alleviate EIF by modulating gut microbiota composition, redox balance, and inflammatory pathways (Cui et al. 2021; Huang et al. 2025).

### 2.3 Mitochondrial Adaptation, Signaling, and Mitophagy

Most anti‐fatigue research has focused on mitigating EIF through energy supplementation, attenuation of oxidative stress, and modulation of central nervous system function, as discussed above. However, these approaches may not adequately address fundamental mechanisms, such as impaired mitochondrial oxidative phosphorylation efficiency, which is now recognized as a critical determinant in the development and regulation of EIF (Yu et al. 2025). A growing body of evidence indicates that certain interventions exert anti‐fatigue effects by promoting mitochondrial biogenesis and enhancing mitochondrial function. For example, *Atractylodes macrocephal*a Koidz water extract has been shown to increase mitochondrial density, ATP content, and endurance in skeletal muscle of mice, accompanied by upregulation of PGC‐1α, NRF1, and TFAM, as well as increased activities of tricarboxylic acid cycle and oxidative phosphorylation enzymes (Ying et al. 2025). Similarly, Maca supplementation modulated skeletal muscle energy metabolism by upregulating mitochondrial biogenesis and function, while reducing exercise‐induced mitochondrial oxidative damage and apoptosis (Orhan et al. 2022; Zhu et al. 2021). In addition, *Brassica rapa* *L.*, aqueous extract enhanced mitochondrial function by modulating the tricarboxylic acid cycle in muscle tissue through the AMPK/PGC‐1α/TFAM signaling pathway. Together with gut microbiota modulation, it may also help maintain intestinal integrity and restrain Gram‐negative bacterial growth, suggesting a dual energy–gut regulatory mechanism (Wang et al. 2023). Collectively, these findings support the concept that nutritional interventions can improve both mitochondrial quantity and quality, thereby enhancing aerobic energy metabolism and alleviating EIF.

Mitophagy mediates selective removal of damaged mitochondria and is crucial for maintaining mitochondrial homeostasis. The extent and timing of mitophagy activation determine whether the outcome is adaptive or maladaptive: moderate activation facilitates quality control, whereas inadequate or excessive mitophagy can compromise mitochondrial networks and exacerbate fatigue (Filler et al. 2014; Yu et al. 2025). In exhaustive exercise mice, *Rhodiola crenulata* extract improved endurance and redox status while suppressing excessive PINK1/Parkin‐dependent mitophagy and restoring mitochondrial morphology and function (Hou et al. 2020). Other natural compounds such as sulforaphane and ginseng extract (ginsenoside‐enriched) modulate mitophagy, thereby attenuating EIF and organ injury in animal models (Ge et al. 2024; Ruhee et al. 2025). Integration of these findings with evidence from *Atractylodes macrocephala* Koidz., *Brassica rapa* *L*., and Maca (Wang et al. 2023; Ying et al. 2025; Zhu et al. 2021) further emphasizes the importance of “mitochondrial quality control” as a key target for nutritional regulation of EIF. However, future studies using multi‐organ, multi‐omics approaches are needed to delineate how nutritional modulation of mitochondrial quality control in different tissues converges on systemic fatigue outcomes.

### 2.4 Gut Microbiota Modulation: From Gut–Muscle to Gut–Brain Axis

Studies on gut microbiota and EIF have expanded rapidly. Dietary polysaccharides are fermented by gut microbes to produce short‐chain fatty acids (SCFAs) such as acetate, propionate, and butyrate, which serve as additional energy substrates, enhance glycogen storage, facilitate lactate clearance, and exert anti‐inflammatory and antioxidant effects (Yang et al. 2025; Zhou et al. 2023). *Paecilomyces hepiali* spores markedly extended swim time and reduced fatigue‐related biomarkers in mice while reshaping gut microbial composition by increasing the relative abundance of *Alistips*, *Eubacterium*, *Bacterium*, *Parasutterella*, and *Olsenella* (Guan et al. 2022). Neoagarotetraose significantly modulated gut microbiome composition and increased fecal SCFA levels and protected against intense exercise‐induced intestinal injury by strengthening barrier function (Zhang et al. 2017). *Astragali Radix–Codonopsis Radix–Jujubae Fructus* water extracts alleviated EIF in mice through coordinated modulation of the gut microbiota and metabolic pathways; four key pathways were determined, including sphingolipid metabolism, glycerophospholipid metabolism, valine, leucine, and isoleucine biosynthesis, and D‐arginine and D‐ornithine metabolism (He, Chen, et al. 2022). Probiotic interventions provide complementary evidence. *Lactobacillus rhamnosus* SDSP202418 decreased lactate and blood urea nitrogen and improved antioxidant status while favorably altering gut microbial composition, subsequently increasing the exhaustion time of the mice (Yang et al. 2024). Live and heat‐treated *Lacticaseibacillus paracasei* NB23 both enhanced mice exercise performance; grip strength and swim endurance were enhanced dose‐dependent and reduced fatigue markers, with live bacteria showing greater effects (Lee et al. 2025). Extruded cereal products with *Cordyceps militaris* and other functional fermented products also improved EIF outcomes via gut microbiota–metabolite–muscle interactions (Cui et al. 2021; Huang et al. 2025; Zhong et al. 2017). More importantly, high‐intensity exercise can increase intestinal permeability, allowing endotoxin translocation into the circulation and triggering systemic inflammation and secondary muscle damage (Zhao et al. 2026). Several polysaccharides and probiotics have been shown to up‐regulate tight‐junction proteins, reduce circulating lipopolysaccharide, and attenuate inflammatory cascades in exercise models, effectively interrupting the detrimental “bacteria–gut–muscle” loop (Cui et al. 2021; Guan et al. 2022; He, Chen, et al. 2022; Yang et al. 2024; Zhang et al. 2017).

Recent research has introduced a “microbiota–gut–brain” axis, further revealing the importance of gut microbiota in regulating central nervous system fatigue (Dohnalová et al. 2022; Zhao et al. 2026). This crosstalk involves complex neural, endocrine, and immunological pathways. Microbial modulation of tryptophan–serotonin metabolism, GABA production, SCFA‐driven neurotrophic signaling, and vagal pathways may influence central fatigue (Cordeiro et al. 2017; Zhao et al. 2026). Nonetheless, an important limitation remains: most studies only report associations between microbial alterations and improvements in fatigue, without delineating a comprehensive causal pathway that links the gut microbiota and its metabolites to the regulation of fatigue‐related physiological processes (Zhao et al. 2026). Overall, gut‐targeted nutritional interventions coordinate energy metabolism, oxidative and inflammatory control, barrier protection, and central regulation, positioning the gut microbiota as a highly attractive systems‐level target for EIF management.

Nutritional interventions encompass a broad spectrum, ranging from classical macronutrient manipulation to functional polysaccharides, polyphenols, herbal extracts, probiotics, and fermented products (Table 1). Despite this heterogeneity, several unifying principles emerge, including optimization of substrate availability, reinforcement of antioxidant and anti‐inflammatory defenses, enhancement of mitochondrial quality control, and restoration of gut microbial homeostasis. Nevertheless, important gaps remain. The current evidence base is dominated by rodent studies, with relatively few well‐designed randomized controlled trials (RCTs) in humans. Rigorous RCTs are required to establish efficacy, define dose–response relationships, and evaluate safety across different exercise modalities and populations. Notably, although mitophagy has been proposed as a central regulatory mechanism in EIF, this mitophagy‐centered model is primarily supported by preclinical evidence, and direct validation in humans remains lacking. In addition, future research should prioritize systems‐level approaches integrating multi‐omics and causal inference frameworks to identify functionally relevant microbial taxa and metabolites and to enable microbiota‐targeted nutritional strategies.

**TABLE 1:** Recent advances of nutritional interventions in exercise‐induced fatigue.

Item	Subjects	Dose	Duration	Exercise protocol	Outcomes	Ref
Rhodiola crenulata oral liquid	Male ICR mice	1.02, 3.03, 6.06 mL/kg‐BW/day; oral gavage	2 weeks	3‐day swim training → weighted exhaustive swim (5% BW tail load; 1 h after last dose)	↓ lactate, CK, LDH, MDA; ↑ SOD, CAT, T‐AOC; ↑ CS, SDH, Na^+^/K^+^‐ATPase; ↑ liver/muscle glycogen; suppressed excessive PINK1/Parkin mitophagy; improved mitochondrial ultrastructure	Hou et al. (2020)
Multi‐ingredient formula (BCAA + β‐alanine + creatine + L‐carnitine + quercetin + betaine)	Mice	Daily total: Val 125, Ile 125, Leu 250, β‐alanine 182, creatine 250, L‐carnitine 200, quercetin 25, betaine 284 mg/kg‐BW; gavage twice/day	3 or 7 days	Exhaustive treadmill test (time/distance to exhaustion as endpoint)	7 days: ↑ time to exhaustion (~+14%) and distance (~+20%); metabolic shift toward fat utilization (AMPK/ACC, CPT1B, CD36); ↓ oxidative stress and muscle injury markers (e.g., MDA, CK, LDH)	Chen et al. (2021)
Fermented deer blood (FDB)	Male C57BL/6J mice	30 or 150 mg/kg‐BW/day (oral)	3 weeks	Incremental treadmill to exhaustion; training every other day	↑ glycogen storage and antioxidant capacity; ↓ metabolic byproduct accumulation and tissue injury markers; remodeled small‐intestine microbiota and metabolites (gut–metabolite mechanisms)	Cui et al. (2021)
α‐Klotho supplementation	Male C57BL/6J mice	0.2 mg/kg‐BW/day, intraperitoneal injection	6 days	6‐day weighted exhaustive swim (5% BW load); grip strength tracked	Restored grip strength; ↓ CK; ↓ H_2_O_2_ and ↑ antioxidant defenses (NRF2/HO‐1, PGC‐1α‐related); promoted hepatic glycogen supercompensation (AKT/GS, GLUT4)	Zheng et al. (2025)
Resveratrol	Sprague–Dawley rats	50 mg/kg‐BW; oral gavage; given 1 h post‐exercise in treatment group	6 weeks	6‐week weighted swim training (5% BW load; 60 min/session; 6 days/week)	↓ BUN and CK; ↓ muscle MDA; ↑ total SOD; ↑ Ca^2+^‐Mg^2+^‐ATPase, Na^+^‐K^+^‐ATPase, SDH, CS; upregulated SIRT1/PGC‐1α/NRF1 pathway (mitochondrial energy metabolism)	Lou et al. (2023)
Trilobatin	Mice	Trilobatin or taurine (1 g/kg‐BW) by gavage	28 days (twice a day)	Rope climbing + exhaustive swim	↓ lactate, CK, BUN; ↑ liver/muscle glycogen; ↑ antioxidant enzymes; activated Nrf2/ARE and inhibited ferroptosis	Xiao et al. (2022)
Astragali Radix–Codonopsis Radix–Jujubae Fructus water extracts (ACJ)	Mice	4.69 or 18.75 g/kg‐BW/day (oral)	6 weeks	Weighted exhaustive swim (5% BW tail load; 40 min after last dose)	↑ endurance and fatigue‐related indices; ↑ liver glycogen; 16S + fecal metabolomics suggest gut microbiota/metabolite pathways (lipids, glycerophospholipids, BCAA biosynthesis, D‐arginine/D‐ornithine metabolism)	He, Chen, et al. (2022)
Phlorizin	Mice	2.5, 5, 10, and 20 mg/kg‐BW (gavage; twice a day)	28 days	Weight‐loaded forced swimming test; rotarod test	↓ BUN, CK, lactate; ↑ liver/muscle glycogen; improved oxidative stress; activated Nrf2/ARE and upregulated HO‐1/NQO1	Ma, Deng, et al. (2022)
Soluble garlic polysaccharide	Male ICR mice	1.25 or 2.5 g/kg‐BW (gavage)	7 weeks	Weight‐bearing swimming test	↑ endurance and fatigue‐related indices; ↑ liver/muscle glycogen; ↑ antioxidant defenses (↑ SOD, GSH‐Px, and CAT); AMPK/PGC‐1α pathway; Modified gut microbiota and metabolites (gut–metabolite mechanisms)	Li et al. (2024)
Ellagic acid	Male ICR mice	1, 75, and 150 mg/kg‐BW (gavage)	6 weeks	Forced swimming test; grip strength test	↑ endurance and grip strength; ↑ liver/muscle glycogen; improved oxidative stress/inflammation indices	Liu et al. (2023)

## 3 Acupuncture‐Based Strategies in EIF: Mechanistic Insights

### 3.1 Complexity of EIF Mechanisms and the Rationale for Traditional Chinese Medicine

Fatigue is a complex process that includes neurotransmitter‐mediated central nervous system fatigue, insufficient energy‐mediated decrease in muscle contraction capacity, and excess ROS‐mediated muscle damage (Ma et al. 2021). Despite detailed descriptions of energy supply failure, ROS overproduction, inflammatory amplification and activation of sympathetic adrenomedullary (SA) system and hypothalamic–pituitary–adrenal (HPA) axis, controlling EIF via pharmaceutical means seems inapplicable currently. Against this background, TCM interventions have attracted growing interest in sports medicine and EIF management (Kang et al. 2021). In TCM theory, EIF is usually categorized under “overstrain”, “consumption”, or “bi‐syndrome”. The core pathogenesis is “depletion of essence–Qi–Shen and disharmony of Yin–Yang and Qi–Blood”, often accompanied by “Qi stagnation, blood stasis, phlegm retention and toxin accumulation”. Lactate, H^+^, inorganic phosphate, urea nitrogen, and free radicals that accumulate during intense exercise can be regarded as “phlegm, stasis, and toxin” obstructing meridians and vessels, impairing the circulation of Qi and Blood. Conceptually, this aligns with modern descriptions of metabolite accumulation, impaired perfusion and free radical–driven inflammatory amplification (Cordeiro et al. 2017; Kang et al. 2021; Liu et al. 2022; Zhao et al. 2026; Zhou et al. 2023). A recent systematic review showed that TCM is beneficial for sports injuries and in enhancing skill development and is becoming increasingly popular among athletes and fitness enthusiasts, supporting its potential relevance to fatigue management (Liu et al. 2022). In sports medicine, TCM techniques such as tuina (manual therapy), cupping, and acupuncture have been incorporated into injury prevention and post‐exercise recovery to alleviate muscle soreness, restore joint function, and facilitate performance recovery (Kang et al. 2021). Evidence from chronic fatigue syndromes and delayed‐onset muscle soreness (DOMS) provides indirect clinical context. Meta‐analyses show that acupuncture and moxibustion significantly improve fatigue scores and quality of life in patients with chronic fatigue syndrome, with only mild adverse events (Wang et al. 2017). In DOMS, systematic reviews and RCTs suggest that acupuncture intervention after intense exercise reduces pain and accelerates recovery of strength (Huang et al. 2020; Hübscher et al. 2008). Cardoso et al. (2020) found that verum acupuncture can reduce DOMS by one‐third. However, these related conditions should not be interpreted as direct EIF‐specific evidence. Among traditional interventions, acupuncture, EA, and TEAS stand out because they are relatively safe, drug‐free, free of addictive or banned substances, simple to administer, and low‐cost (Kang et al. 2021). They can be delivered in training facilities and competition venues and are well suited for athletes and fitness enthusiasts (Kang et al. 2021; So et al. 2007).

### 3.2 Acupuncture, Electroacupuncture, and TEAS in EIF

A systematic review of clinical trials reported that acupuncture after intense exercise effectively reduced DOMS and enhanced muscle recovery; the long‐lasting effect of acupuncture intervention on DOMS started from 24 h and would reach a peak at the time point of 72 h post exercise (Huang et al. 2020). RCTs further showed that needling at specific acupoints reduces post‐exercise muscle pain, improves isometric strength, and accelerates recovery (Cardoso et al. 2020; Hübscher et al. 2008). A systematic review indicates that electroacupuncture (EA) activates multiple antinociceptive and anti‐stress pathways, including the release of opioid peptides, activation of descending serotonergic and noradrenergic inhibitory systems, and modulation of adenosine and endocannabinoid signaling in peripheral and central nervous systems (Zhang et al. 2014). These neurohumoral changes explain not only analgesic effects but also the capacity of acupuncture strategies to influence peripheral and central components of EIF.

EA may provide stronger analgesia than manual acupuncture; and electrical stimulation via skin patch electrodes is as effective as EA (Ulett et al. 1998; Zhang et al. 2014). In addition, EA (particularly at low frequency) produced more widespread functional MRI signal increase than manual acupuncture did (Napadow et al. 2005). Hence, EA generally applies low‐ or medium‐frequency (2–10 Hz) electrical stimulation through inserted acupuncture needles. It stabilizes and controls the intensity and frequency of stimulation, improving reproducibility and often enhancing analgesic and neuro‐modulatory effects (Zhang et al. 2014). Experimental and clinical studies show that EA can produce stronger and longer‐lasting analgesia than manual acupuncture, which blocks pain by activating a variety of bioactive chemicals through peripheral, spinal, and supraspinal mechanisms (Zhang et al. 2014). These properties make EA a useful tool to modulate central arousal, emotional state, and autonomic balance in EIF.

Transcutaneous acupoint electrical stimulation (TEAS) delivers electrical stimulation via surface electrodes placed over acupoint regions, thereby activating acupoint‐related nerve endings without needle insertion. Therefore, TEAS is minimally invasive and well suited for application in sports settings (So et al. 2007). Zhou et al. (2025) have summarized six major clinical applications of TEAS, including analgesia, regulation of gastrointestinal function, improvement of reproductive function, enhancement of cognitive function, promotion of limb function recovery, and alleviation of fatigue. In healthy young adults undergoing isokinetic knee fatigue protocols, TEAS applied to selected acupoints—[Zusanli (ST36), Chenshan (BL57), Yanglingquan (GB34), and Sanyinjiao (SP6)]—has been shown to enhance quadriceps strength recovery, reduce perceived fatigue, and shorten recovery time between repeated high‐intensity bouts (So et al. 2007). In clinical populations outside EIF, TEAS applied to bilateral ST36, bilateral Geshu (BL17), and unilateral Qihai (CV6) significantly reduced chemotherapy‐related fatigue in patients with cancer, with the most pronounced effects observed at the P3 time point (Hou et al. 2017). Furthermore, a meta‐analysis of nine randomized controlled trials involving 924 cancer patients demonstrated that TEAS significantly alleviates cancer‐related fatigue, depression, and anxiety, while improving overall quality of life (He, Yuan, et al. 2022). These non‐EIF findings support biological plausibility and broader fatigue‐related relevance, but they should not be interpreted as direct EIF evidence. More recently, a review emphasized that TEAS is safe and well tolerated, with broad applications in analgesia, sedation and fatigue relief in perioperative and chronic disease settings (Zhou et al. 2025). Compared with traditional acupuncture, TEAS is non‐invasive, highly acceptable, and easy to deploy immediately before or after training and competition—features that are particularly advantageous in sports field (So et al. 2007). Several studies suggest that TEAS at ST36 and other classical “anti‐fatigue” acupoints lowers post‐exercise blood lactate and creatine kinase, regulates endocrine function and cell apoptosis, thereby attenuating EIF (So et al. 2007; Zhou et al. 2025). Together, these findings support TEAS as a practical and effective strategy in EIF‐related recovery settings, although direct EIF‐specific evidence remains limited.

### 3.3 Mechanisms by Which Acupuncture Alleviates EIF

Interventional trials demonstrate that needling at Shenmen (HT7) increases very‐low‐frequency, low‐frequency, and high‐frequency components of heart rate variability (HRV), reflecting a shift toward parasympathetic dominance (Huang et al. 2015). Although HRV evidence is not uniformly EIF‐specific, it suggests a plausible autonomic mechanism relevant to post‐exercise recovery. In EIF, such autonomic rebalancing may help dampen prolonged sympathetic activation and cardiovascular strain after heavy exercise and thus reduce central fatigue. Classical analgesic mechanisms of acupuncture, including the release of endorphins, enkephalins, and dynorphins in the brain and spinal cord and activation of descending serotonergic and noradrenergic pathways, also contribute to reduced post‐exercise pain (Zhang et al. 2014). EA can down‐regulate corticosterone, adrenocorticotropic hormone, and pro‐inflammatory cytokines under stress conditions, and modulate the HPA axis (Fan 2023). In addition, EA inhibits nociceptive signaling at peripheral, spinal, and supraspinal levels in chronic pain models, with clear frequency dependence (Zhang et al. 2014). These findings suggest that acupuncture‐based strategies exert systemic effects on the SA system and HPA axis and support their possible relevance to EIF.

At the peripheral level, acupuncture and EA influence local muscle blood flow and metabolism. Clinical studies in DOMS indicate that acupuncture improves microcirculation in injured muscle, accelerates clearance of lactate and other metabolites, and in some trials, enhances recovery of isometric strength and functional performance (Cardoso et al. 2020; Huang et al. 2020; Hübscher et al. 2008; Sandberg et al. 2005). Similarly, TEAS applied to ST36 and other lower‐limb acupoints shortens the time required for strength recovery and reduces perceived fatigue following repeated high‐intensity exercise, consistent with improved local perfusion and metabolic recovery (So et al. 2007). Evidence from animal models provides additional mechanistic insights, although important distinctions between experimental contexts should be noted. In acute exercise models (e.g., forced swimming), acupuncture and EA have been shown to increase mitochondrial Ca^2+^‐ATPase activity and antioxidant enzyme capacity in skeletal muscle, thereby supporting mitochondrial function and delaying fatigue onset (Gao et al. 2008). In contrast, studies in non‐EIF pathological or injury models—including type 2 diabetes and muscle injury—demonstrate that EA can activate the AMPK/PGC‐1α/TFAM pathway, improve glucose and lipid metabolism, and modulate gene expression related to inflammation, apoptosis, and tissue remodeling (Liu, Guo, et al. 2021; Luo et al. 2025). However, these models differ fundamentally from EIF in terms of pathophysiology. Therefore, while such findings support a potential role for acupuncture‐based interventions in regulating mitochondrial function and cellular stress responses, they should not be interpreted as direct evidence of EIF‐specific mechanisms. Instead, they provide mechanistic plausibility that requires further validation in well‐designed EIF‐focused studies. Taken together, current evidence suggests that acupuncture‐based strategies may extend beyond improving microcirculation and metabolite clearance to influence mitochondrial function and skeletal muscle adaptation (Gao et al. 2008; Liu, Guo, et al. 2021; Luo et al. 2025). These effects partially parallel those of nutritional interventions targeting mitochondrial metabolism, although the extent to which these shared pathways operate in EIF remains to be established.

Increasing evidence shows that acupuncture and EA activate the nuclear factor erythroid 2‐related factor 2 (Nrf2)–antioxidant response element pathway and inhibit NF‐κB, thereby exerting antioxidant and anti‐inflammatory effects (Huang and Hsieh 2021; Yu et al. 2014). In a rabbit model of endotoxic shock‐induced acute lung injury, EA at ST36 and Feishu (BL13) increased pulmonary Nrf2 and heme oxygenase‐1 expression, reduced malondialdehyde levels, and enhanced antioxidant enzymes activities, leading to less oxidative damage and tissue injury (Yu et al. 2014). Meanwhile, in a mouse model of metabolic dysfunction‐associated fatty liver disease, EA improved intestinal barrier structure and restored gut–liver axis homeostasis by regulating the α7nAChR–HO‐1/p38 MAPK/NF‐κB pathway, reducing TNF‐α, IL‐1β, and other pro‐inflammatory cytokines (Wang et al. 2025). In hepatic ischemia–reperfusion injury, EA pretreatment at ST36 markedly demonstrated a protective effect on hepatic ischemia–reperfusion injury in mice by alleviating oxidative stress, hepatocyte death, and inflammation response via regulating Nrf2 pathway (Jiang et al. 2023). While these studies consistently demonstrate that acupuncture can attenuate oxidative stress and inflammatory responses via activation of Nrf2 signaling and inhibition of NF‐κB pathways, they should be interpreted as indirect evidence rather than direct mechanistic validation in EIF.

Collectively, acupuncture‐based strategies are widely applied in diverse fields, including sports medicine and the treatment of various diseases, because they are relatively safe, drug‐free, and simple to administer (Zhang et al. 2014; Zhou et al. 2025). They provide multi‐dimensional regulation of neuroendocrine function, autonomic balance, skeletal muscle metabolism, and oxidative–inflammatory networks, thereby addressing both peripheral and central components of fatigue (Huang and Hsieh 2021; Yu et al. 2014). EA and TEAS render stimulation parameters (frequency, intensity, duty cycle) more controllable and reproducible than manual acupuncture and can be integrated into modern training and rehabilitation programs (So et al. 2007; Zhang et al. 2014; Zhou et al. 2025). However, several limitations should be acknowledged. Clinical studies of EA and TEAS show wide heterogeneity in acupoint selection, needling depth, manual manipulation, stimulation frequency and intensity, and treatment duration, which hampers cross‐study comparison, meta‐analysis, and the development of standardized protocols for EIF. Acupoint prescriptions remain largely experience‐based; although ST36, SP6, and a few other “classical anti‐fatigue” points are frequently used, RCTs systematically comparing different acupoint combinations or dosing strategies are scarce (Kang et al. 2021; So et al. 2007; Zhou et al. 2025). Many EIF‐related trials focus on short‐term outcomes and single endpoints (e.g., subjective fatigue or blood lactate) and lack high‐quality RCTs using performance, recovery kinetics, or long‐term adaptation as primary outcomes (Table 2).

**TABLE 2:** Recent advances of acupuncture‐based strategies in exercise‐induced fatigue.

Item	Subjects	Dose	Duration	Exercise protocol	Outcomes	Ref
Moxibustion at CV8 (Shenque)	Male Sprague–Dawley rats	Moxibustion at CV8, 15 min/session; post‐exercise	12 weeks	12‐week treadmill training to establish EIF model	Prevented fatigue‐associated cardiac structural changes and improved cardiac function; minimal effects in non‐exercise controls (suggesting relative safety)	Shen et al. (2021)
Moxibustion at CV8 (Shenque)	Sprague–Dawley rats	CV8 moxibustion 15 min/session; “5 days on +2 days off” cycle; total ~40 sessions	8 weeks	8‐week treadmill long‐term fatigue model	Improved general condition and immune indices; ↓ IL‐6/IFN‐γ/TNF‐α; inhibited NF‐κB/NLRP3/caspase‐1 inflammatory pathway	Zhang et al. (2025)
Acupoint catgut embedding	Sprague–Dawley rats	Catgut embedding at acupoints (Qiang Feng, Bai Hui., Xie Qi, Han Gou, Yang Wa, and Qian Shen)	8 weeks	7‐week treadmill fatigue model	Transcriptome + metabolomics suggest improved endurance via lipid metabolism regulation; implicated PPAR signaling; ↓ lactate accumulation and improved injury‐related markers	Song et al. (2023)
Press‐tack needle acupuncture (PC6 + ST36)	Sports students	Press‐tack needles at PC6 and ST36; applied 30 min before exercise	Single session	High‐intensity exercise; blood lactate measured at 5‐ and 30‐min post‐exercise	Greater lactate declines from 5 → 30 min vs. control; suggests faster post‐exercise lactate clearance/recovery	Djaali et al. (2023)
Acupuncture assessed by transcranial magnetic stimulation (TMS)	Regular exercisers	Acupuncture vs. sham	Single session	Exercise‐induced fatigue followed by TMS measures	Faster recovery in corticospinal excitability metrics (↑ MEP amplitude, ↓ latency) and heart rate recovery; lactate differences not significant	Hu et al. (2024)
Acupuncture for delayed onset muscle soreness (DOMS)	Healthy volunteers	Verum acupuncture at ST34, ST36 and LR3	Acute/short‐term	Strenuous exercise inducing DOMS (EIMD protocol)	Verum acupuncture can reduce the occurrence of acute muscle soreness by one‐half and delayed onset muscle soreness by one‐third	Cardoso et al. (2020)
Immediate acupuncture effects on force and DOMS	Healthy volunteers	Acupuncture at IG4, IG11, E36, and VB34; lasted for ~20 min	Single/short follow‐up	DOMS/force recovery testing	A single acupuncture session has immediate effects on muscle activation; acupuncture provided an analgesic effect that was able to modulate pain rating and threshold	Antonassi et al. (2021)
Systemic acupuncture and strength/power in athletes	Male handball players	Acupuncture at GV20, LI15, LU5, LI4, LR13, ST36, GB34, SP6 for 30 min	Single session	Strength/power tests (lower limb power, grip strength, and maximal isometric voluntary contraction)	An acute systemic acupuncture treatment provided no influence on MIVC and lower limb power	Ferreira et al. (2024)
TEAS for quadriceps fatigue recovery	Healthy young adults	TEAS at ST36, BL57, GB34, SP6	Single session	Isokinetic knee fatigue exercise with repeated bouts	Improved quadriceps strength recovery, reduced subjective fatigue, shortened recovery time	So et al. (2007)

## 4 Mechanistic Convergence and Integrative Perspectives

### 4.1 Shared Biological Targets and Key Mitophagy Pathways

Synthesizing evidence from the nutritional and acupuncture‐based literature, several biological targets have been discussed as potentially relevant to EIF. First, the AMPK/PGC‐1α axis. Nutritional interventions have been reported to influence AMPK and its co‐activator PGC‐1α, and may thereby be associated with mitochondrial biogenesis, tricarboxylic acid cycle substrate availability, and ATP production, with possible implications for endurance and recovery (Wang et al. 2023; Ying et al. 2025; Zhu et al. 2021). EA and acupuncture have also been reported to affect AMPK/PGC‐1α/TFAM signaling in skeletal muscle and other tissues, and may be associated with changes in glucose and lipid metabolism, ATP content, and mitochondrial biogenesis or function (Gao et al. 2008; Liu, Guo, et al. 2021; Luo et al. 2025). Second, the Nrf2 and NF‐κB signaling pathways. Antioxidant nutrients such as vitamins C and E (Kim et al. 2022), gastrodin (Zhang et al. 2023), trilobatin (Xiao et al. 2022), and sulforaphane (Ruhee et al. 2025) have been linked to activation of Nrf2/ARE signaling and inhibition of NF‐κB, and may therefore influence exercise‐induced oxidative stress and inflammatory responses. Similarly, acupuncture and EA have been reported to up‐regulate Nrf2 and HO‐1 and to inhibit NF‐κB nuclear translocation in acute lung injury, metabolic disease, and ischemia–reperfusion models, with accompanying reduction in ROS and inflammatory cytokines (Jiang et al. 2023; Wang et al. 2025; Yu et al. 2014). Third, cytokine and immune networks. Nutritional strategies have been associated with modulation of IL‐1β, IL‐6, TNF‐α, and other cytokines, which may be relevant to low‐grade inflammation and immune disturbances induced by strenuous exercise (Wang et al. 2021; Xiong et al. 2020; Zhou et al. 2023). EA, through vagal–adrenal pathways, α7nAChR signaling, and local immune regulation, has also been linked to changes in cytokine profiles (Fan 2023; Jiang et al. 2023; Liu, Wang, et al. 2021). Overall, nutritional and acupuncture‐based interventions appear to involve partially overlapping pathways, including AMPK–PGC‐1α, Nrf2–NF‐κB, and cytokine‐related networks; however, this mechanistic overlap should not be interpreted as evidence supporting additive, synergistic, or clinically validated integrated interventions in EIF.

Given the central role of mitochondria in cellular energy homeostasis and the role of mitophagy in the selective removal of damaged mitochondria, mitophagy is likely relevant to the maintenance of cellular energy balance and viability. By facilitating the clearance of damaged mitochondria, mitophagy may also contribute to redox homeostasis and cellular stress adaptation (Yu et al. 2025). However, the role of mitophagy in EIF is unlikely to be uniformly beneficial. Moderate and well‐coordinated mitophagy may support mitochondrial quality control and recovery, whereas excessive or dysregulated mitophagy could contribute to mitochondrial depletion and impaired ATP supply. In addition, these responses are likely to be tissue‐specific, with potentially different thresholds and functional consequences across skeletal muscle, neural tissues, and other metabolically active organs (Yu et al. 2025). Accordingly, mitophagy has emerged as a relevant mechanistic target in EIF research. Further clarification of mitophagy‐related pathways in EIF may help refine mechanistic understanding and inform future intervention studies. Exercise, hypoxia, oxidative stress, and ischemia can all activate AMPK‐mediated autophagy. Elevated AMP levels activate AMPK, which in turn activates its downstream target, Unc‐51‐like autophagy‐activating kinase 1 (ULK1). At the same time, the autophagy inhibitor mTORC1 is suppressed, thereby promoting mitophagy (Memme et al. 2021). PTEN‐induced kinase 1 (PINK1) accumulates on damaged mitochondrial membranes and recruits the E3 ubiquitin ligase Parkin to initiate mitophagy. PINK1 activity peaks approximately 12 h after exercise, coinciding with the time point at which mitochondrial damage is maximal (Yu et al. 2025). Nix and BNIP3 are members of the Bcl‐2 family. Hypoxia upregulates Nix and BNIP3 expression, thereby inducing mitophagy; in addition, BNIP3 regulates other mitophagy pathways by inhibiting PINK1 degradation (Zhang et al. 2008). FUNDC1 is an outer mitochondrial membrane receptor that senses hypoxia and initiates mitophagy through dephosphorylation followed by binding to LC3 (Wu et al. 2016). Nevertheless, whether activation of these pathways is ultimately adaptive or maladaptive likely depends on the magnitude and duration of stress as well as the tissue context.

### 4.2 A Conceptual Integrative Model

**FIGURE 1:**
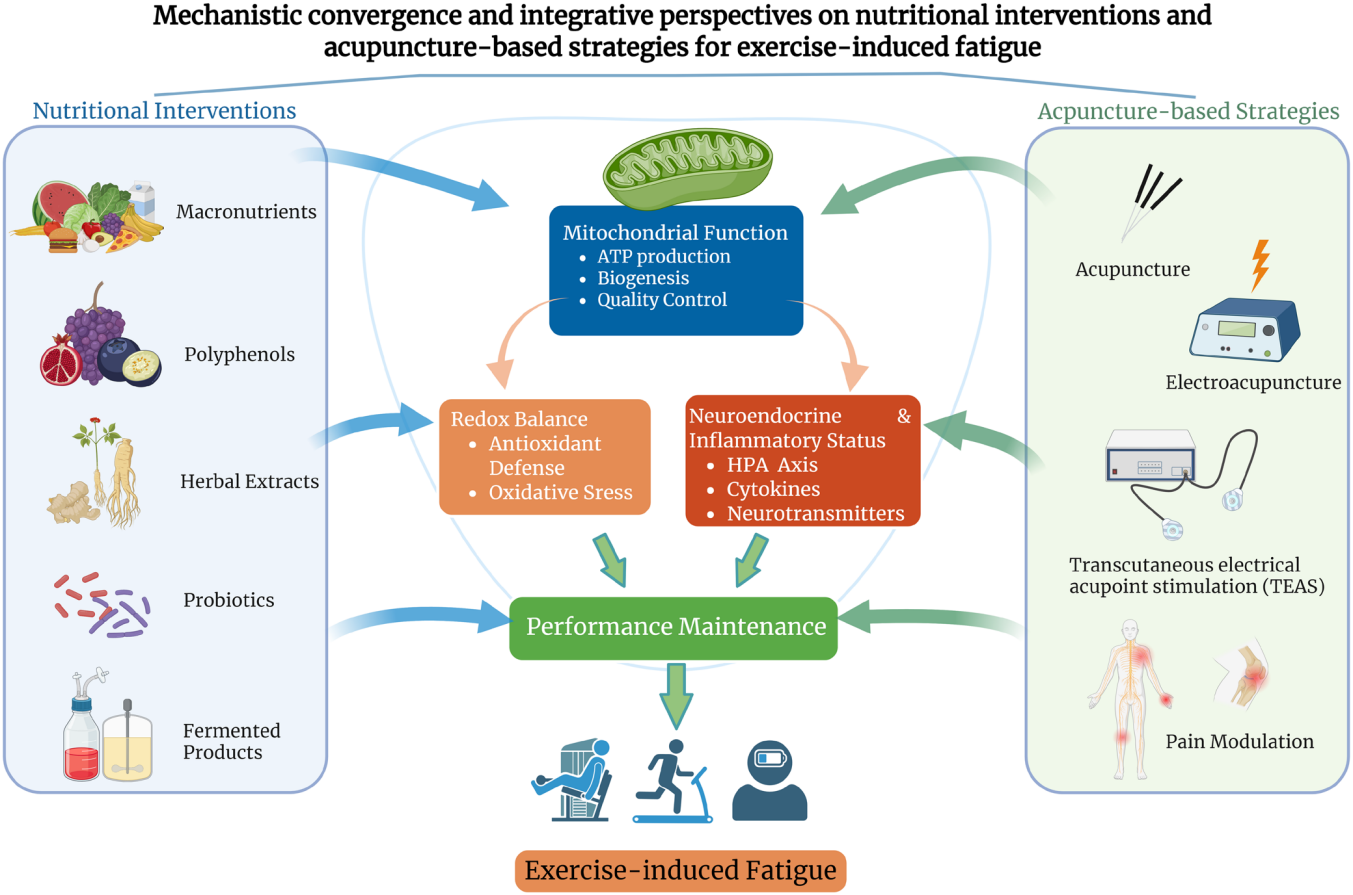
Conceptual summary of the main mechanistic domains discussed for nutritional interventions and acupuncture‐based strategies in exercise‐induced fatigue. Nutritional interventions are mainly linked to substrate availability and redox balance, whereas acupuncture‐based strategies are mainly linked to neuroendocrine and inflammatory regulation. Both have been associated with mitochondrial function and performance‐related recovery processes. The figure is conceptual and does not imply proven additive or synergistic efficacy.

Nutritional interventions are primarily discussed in relation to substrate availability and redox balance. Adjustments in micronutrient intake, exercise‐related timing, and the use of selected functional compounds may influence glycogen metabolism, fatty acid utilization, and intracellular antioxidant capacity and therefore may be relevant to energy deficit and redox imbalance in EIF. Acupuncture‐based strategies are more commonly discussed in relation to neuroendocrine and inflammatory regulation. By affecting autonomic balance, HPA axis activity, and the vagal–adrenal axis, they may influence sympathetic activity and systemic inflammatory tone. Pathways such as Nrf2/NF‐κB and α7nAChR have been implicated as possible mediators of these effects. Both approaches have been linked, to varying degrees, to mitochondrial efficiency and recovery kinetics (Figure 1). Nutritional interventions have been reported to affect mitochondrial biogenesis and fatty acid oxidation, possibly through AMPK/PGC‐1α signaling, whereas EA and TEAS may also modulate mitochondrial antioxidant defenses and ATP production via partly overlapping pathways. During recovery, nutritional support may help maintain substrate availability and antioxidant capacity, while acupuncture‐based strategies may contribute to recovery from high‐stress states toward homeostasis through neuroendocrine and inflammatory modulation.

Although no high‐quality RCTs have directly compared combined “nutrition + EA/TEAS” with single interventions (Vittorio et al. 2020), the following considerations should be regarded as tentative mechanistic propositions rather than evidence of therapeutic complementarity. From a temporal perspective, nutritional strategies are often discussed in relation to pre‐, intra‐, and post‐exercise metabolic support, as well as longer‐term day‐to‐day recovery management (Aragon and Schoenfeld 2013), whereas acupuncture and TEAS have also been examined in settings immediately before or after training or competition, particularly in relation to neuroendocrine state, pain, sleep, and mood regulation (Kang et al. 2021; Ulett et al. 1998). Likewise, nutritional interventions are more commonly associated with longer‐term adaptations, such as changes in body composition and mitochondrial function, whereas acupuncture‐based interventions are more often studied in relation to more immediate physiological and symptomatic responses, including HRV, acute pain, and inflammatory fluctuations (Lee et al. 2010; Liu et al. 2022; Yu et al. 2025). At the mechanistic level, nutritional interventions are primarily discussed in relation to metabolic and immune pathways, with possible indirect effects on central nervous system function (Dohnalová et al. 2022; Zhao et al. 2026), while acupuncture‐based strategies are generally understood to begin with neural regulation and may secondarily influence metabolism and immunity through pathways such as the brain–gut–muscle and brain–adrenal axes (Sun et al. 2023). If studied together, these approaches might influence both ‘top‐down’ (neuroendocrine) and ‘bottom‐up’ (metabolic–immune) pathways, but this possibility remains hypothetical. From a conceptual standpoint, EA and TEAS are more often discussed in relation to short‐term neuroendocrine and symptom modulation, whereas nutritional strategies are more often discussed in relation to longer‐term metabolic support and recovery adaptation. It is important to emphasize that these hypotheses remain conceptual and should not be interpreted as evidence of additive or supra‐additive efficacy. There is currently no robust clinical evidence demonstrating that combined nutritional and acupuncture‐based interventions improve performance or recovery beyond single interventions. Therefore, it is more precise to describe them as showing partial mechanistic overlap and possible complementarity, rather than proven additive or synergistic efficacy.

## 5 Conclusions and Future Perspectives

EIF is a complex syndrome associated with disturbances in energy metabolism, oxidative stress, inflammatory responses, and neuroendocrine–immune networks, with emerging contributions from mitochondrial quality control and the gut microbiota. Nutritional interventions are widely discussed as a supportive component of EIF management because they may influence substrate availability, antioxidant and anti‐inflammatory capacity, mitochondrial adaptation, mitophagy‐related processes, and gut microbial composition. Acupuncture‐based strategies represent an additional non‐pharmacological approach that has been associated with multi‐target modulation of neuroendocrine, autonomic, metabolic, and immune systems, and may have relevance to both peripheral and central aspects of fatigue. The convergence of these approaches at key nodes such as AMPK–PGC‐1α, Nrf2–NF‐κB, and cytokine networks indicates partial mechanistic overlap between the two intervention categories, but does not establish additive or synergistic clinical benefit of combined use in EIF. However, the current evidence base remains limited by heterogeneous protocols, small sample sizes, and sparse mechanistic validation, and any synergistic effects of combined nutritional and acupuncture‐based interventions remain unproven. At present, evidence from human studies mainly supports feasibility and some short‐term recovery‐related outcomes, whereas much of the mechanistic detail derives from animal studies or non‐EIF disease models; any advantage of combined use therefore remains a conceptual hypothesis rather than an established clinical finding. Future research should prioritize well‐designed, standardized, multi‐omics and multi‐endpoint trials to clarify the indications and boundaries of these strategies and to directly test whether combined approaches provide benefit beyond single interventions in different sports and populations. Conceptually, the joint consideration of nutritional and acupuncture‐based interventions may offer a useful framework for hypothesis generation regarding network‐based and individualized regulation of EIF. At present, however, this possibility remains speculative and requires rigorous empirical validation.

## Author Contributions

**Yaqin Yang:** investigation, writing – original draft, visualization, data curation. **Lijuan Zou:** data curation, visualization. **Weiyu Lu:** visualization, investigation. **Huashan Pan:** writing – review and editing, methodology, validation. **Yao Yao:** writing – original draft, visualization. **Jiazhou Li:** investigation, formal analysis.

## Funding

This work was funded by the Guangdong Basic and Applied Basic Research Foundation (General Program) (Grant 2024A1515012151); Special and Innovative Project of General Higher Education Institutions in Guangdong Provincial Department of Education (Grant 2025KTSCX303); High‐level Fund Cultivation Program, Guangdong Food and Drug Vocational College (Grant 2025ZR05); and Natural Science Projects (Department‐level Project Cultivation Program) of Guangdong Food and Drug Vocational College (Grant 2025ZR11).

## Conflicts of Interest

The authors declare no conflicts of interest.

## Data Availability

The data that support the findings of this study are available from the corresponding author upon reasonable request.
